# Evidence for Nitric Oxide Synthase Activity in *Staphylococcus xylosus* Mediating Nitrosoheme Formation

**DOI:** 10.3389/fmicb.2017.00598

**Published:** 2017-04-06

**Authors:** Geoffrey Ras, Véronique Zuliani, Patrick Derkx, Tim M. Seibert, Sabine Leroy, Régine Talon

**Affiliations:** ^1^Université Clermont Auvergne – INRA, MEDISClermont-Ferrand, France; ^2^CHR. HANSEN SASSaint Germain les Arpajon, France; ^3^CHR. HANSEN A/SHørsholm, Denmark; ^4^CHR. HANSEN GmbHPohlheim, Germany

**Keywords:** nitric oxide synthase, nitric oxide, nitrosoheme, *Staphylococcus xylosus*, coagulase-negative *Staphylococcus*, oxidative stress

## Abstract

*Staphylococcus xylosus* is used as a starter culture in fermented meat products and contributes to color formation by the reduction of nitrate to nitrite. Nitrite is a food additive that is chemically turned to nitric oxide (NO) in meat but its safety has been questioned. The objective of this study was to determine the ability of NO synthase (NOS) of *S. xylosus* C2a to produce NO. For this purpose, a *nos* deletion mutant (Δ*nos*) in *S. xylosus* was constructed and NO production was evaluated in a test based on its ability to form nitrosomyoglobin and nitrosoheme. Production of NO was abrogated in the Δ*nos* mutant under aerobic conditions and reduced about 35-40% comparing to the wild type C2a under limited oxygenation. This mutant was sensitive to oxidative stress. The expression of genes encoding catalase was modulated in the mutant with an up-regulation of *katA* and a down-regulation of *katB* and *katC*. The Δ*nos* mutant displayed high colony pigmentation after prolonged growth on agar medium. Finally, the Δ*nos* mutant showed no growth in minimal medium. Growth was not restored in the minimal medium by complementation with *nos*, but was restored by either addition of phenylalanine or complementation with *pdt*, a gene that encodes a prephenate dehydratase involved in phenylalanine biosynthesis and co-transcribed with *nos*. Our findings clearly demonstrate NOS-mediated NO production in *S. xylosus*, a meat-associated coagulase-negative *Staphylococcus*.

## Introduction

*Staphylococcus xylosus*, a coagulase-negative *Staphylococcus* (CNS), is commonly used as starter culture to boost color development of cured meat products such as dried fermented sausages (Talon and Leroy, 2006). The typical reddish color relies on the presence of the curing salts, nitrate and nitrite. Nitrate salts are reduced to nitrite by nitrate reductase activity of *S. xylosus* (Talon et al., 1999; Gøtterup et al., 2008). Nitrite is subsequently converted by chemical reactions to nitric oxide (NO), which is able to bind to the ferrous heme-iron to form the stable red nitrosomyoglobin pigment (Gøtterup et al., 2007, 2008).

The use of the curing agents nitrate and nitrite is regulated by law, with specific indications in the United States (Code of Federal Regulations, 2016) and Europe (Commission Regulation [EU], 2011). Nowadays, the safety regarding the use of such additives on meat products is questioned. Nitrites can react with secondary amine groups of muscle proteins as well as compounds present in the gastrointestinal tract to form undesired N-nitroso compounds such as nitrosamines, which could pose a health threat (Honikel, 2008). The meat industry is therefore looking for alternatives to decrease nitrate/nitrite in the production of meat products.

*Staphylococcus xylosus* has been shown to convert metmyoglobin to nitrosomyoglobin in culture medium, in salami (Morita et al., 1998) and in raw meat batter (Li et al., 2013, 2016), without addition of nitrate or nitrite. NO production has been suggested to be linked to NO synthase (NOS) activity. NOS catalyzes the production of NO from l-arginine and was initially described in mammals (Alderton et al., 2001). Homologs of the oxygenase domain of mammalian NOS were identified in Gram-positive bacteria. NOS is involved in the production of NO by *Bacillus subtilis* (Gusarov and Nudler, 2005; Gusarov et al., 2008), *Bacillus anthracis* (Shatalin et al., 2008), *Bacillus cereus* (Montgomery et al., 2010), *Geobacillus stearothermophilus* (Kabir et al., 2008), *Streptomyces turgidiscabies* (Kers et al., 2004), *Deinococcus radiodurans* (Patel et al., 2009), and *Staphylococcus aureus* (van Sorge et al., 2013). *S. aureus* NOS is involved in resistance to oxidative stress and antibiotics, and virulence (Vaish and Singh, 2013; van Sorge et al., 2013; Sapp et al., 2014). In all sequenced *S. aureus* genomes, the *nos* gene is part of a cluster containing another gene encoding a prephenate dehydratase, which is involved in phenylalanine biosynthesis. This genetic organization appears to be unique to staphylococcal genomes (Sapp et al., 2014). The *nos* genes have been identified in many staphylococci (van Sorge et al., 2013; Sánchez-Mainar et al., 2014; Sapp et al., 2014), but NOS-mediated NO production has only been characterized in the pathogenic *S. aureus*. The contribution of the NOS activity of *S. xylosus* to formation of nitrosomyoglobin remains to be demonstrated.

In an attempt to determine the ability of *S. xylosus* NOS to produce NO, we generated a *nos* deletion mutant in the *S. xylosus* C2a strain. NO production in *S. xylosus* C2a and mutants was investigated using an assay based on the conversion of metmyoglobin to red pigment derivatives followed by an extraction of nitrosoheme. We also assessed the sensitivity of the mutants to oxidative stress.

## Materials and Methods

### Bacterial Strains and Growth Conditions

The bacterial strains and plasmids used in this study are listed in **Table 1**. Experiments were performed using *S. xylosus* strain C2a or its isogenic mutants. *S. xylosus* was routinely cultured in Tryptic Soy Broth (TSB, Difco) under aerobic conditions (1:10 volume to flask ratio, 150 rpm) or on Tryptic Soy Agar (TSA, Difco) at 30°C. When required, strains were grown in a minimal medium (MX) as already described (Fiegler and Brückner, 1997). The MX medium, when needed, was supplemented with amino acids (100 mg/L). For genetic manipulation, *Escherichia coli* was cultured in Luria–Bertani (LB, Difco) broth with shaking aeration (150 rpm) or on LB agar (Difco) at 37°C. Antibiotics, when required to maintain plasmids or select recombinants, were added at the following concentrations: for *E. coli*, 100 μg/mL ampicillin; for *S. xylosus*, 20 μg/mL chloramphenicol, or 2.5 to 10 μg/mL erythromycin. All chemicals were from Sigma–Aldrich.

**Table 1:** List of strains and vectors used in this study.

Strain or plasmid	Relevant characteristics	Source or reference
***Staphylococcus xylosus***		
C2a	Derived from the type strain DSM20267 and genetically transformable strain	Götz et al., 1983, LN554884
Δ*nos*	Isogenic mutant of C2a deleted of the SXYL_00923 gene, Δ*nos::ermB*	This study
Δ*nos*pRB*nos*	Δ*nos* complemented with *nos*	This study
Δ*nos*pRB*pdt*	Δ*nos* complemented with *pdt* (SXYL_00922)	This study
Δ*nos*pRB*nospdt*	Δ*nos* complemented with *nospdt*	This study
C2apRB473	C2a containing empty vector pRB473	This study
C2apRB474	C2a containing empty vector pRB474	This study
Δ*nos*pRB473	Δ*nos* containing empty vector pRB473	This study
Δ*nos*pRB474	Δ*nos* containing empty vector pRB474	This study
***Escherichia coli***		
TOP10	Competent strain for plasmid transformation	Invitrogen
**Plasmids**		
pBT2	*E. coli*-*S. xylosus* thermosensitive shuttle vector, Ap^R^ Cm^R^	Brückner, 1997
pEC4	pBluescript KS + derivative. Source of *ermB* gene (EmR). ApR	Brückner, 1997
pRB473	Shuttle vector Ap^R^ and Cm^R^	Brückner, 1992
pRB474	Shuttle vector Ap^R^ and Cm^R^	Brückner, 1992
pBTΔ*nos*	pBT2, SXYL_00923 [13-317]::ermB shuttle vector	This study
pRB*nos*	pRB473 derivate for expression of *nos*	This study
pRB*pdt*	pRB474 derivate for expression of *pdt*	This study
pRB*nospdt*	pRB473 derivate for expression of *nos* and *pdt*	This study

To compare growth kinetics under microaerobic conditions, overnight cultures of *S. xylosus* were diluted to an optical density (OD) of 0.05 to 0.10 at 600 nm and incubated in 100-well microtiter plates with shaking at 30°C in a Bioscreen C plate reader (Labsystems France) while the turbidity was monitored every 30 min for 24 h. Three independent experiments were done for each set of conditions.

### DNA Manipulation

Genomic DNA from *S. xylosus* was prepared from overnight cultures. Briefly, cells were resuspended in Tris-EDTA-sucrose buffer containing 0.1 mg/mL lysostaphin (Sigma-Aldrich) and incubated for 30 min at 37°C. Cells were lysed with sodium dodecyl sulfate and treated with RNase A. Following extraction with phenol-chloroform-isoamyl alcohol (25/24/1) and chloroform, DNA was precipitated with ethanol and resuspended in Tris-EDTA buffer (pH 8.0). Plasmid DNA from *E. coli* was isolated using the NucleoSpin Plasmid Quick-Pure kit (Macherey-Nagel). Restriction digests were performed using high-fidelity restriction enzymes (New England Biolabs). Ligations were performed using T4 DNA ligase (Roche). Amplifications were performed using Phusion High-Fidelity DNA Polymerase (New England Biolabs) for cloning and GO Taq DNA polymerase (Promega) for standard purposes. PCR products were visualized by 0.8% agarose gel electrophoresis and ethidium bromide staining, and imaged on a Gel Doc 2000 (Bio-Rad). As required, they were purified using the QIAquick Gel Extraction kit (Qiagen). DNA sequencing was performed by GATC Biotech (Mulhouse, France).

### Construction of *S. xylosus* NO Synthase Mutant

The oligonucleotides used in this study were designed based on the genome sequence of *S. xylosus* C2a (GenBank accession no. LN554884) and are listed in **Table 2**. A chromosomal *nos* disruption mutant was constructed by deletion of the *nos* coding sequence corresponding to amino acids 11 to 345 (out of 354) and insertion of an erythromycin resistance cassette (*ermB*) using the temperature-sensitive vector pBT2. Briefly, a 756-bp upstream fragment and a 783-bp downstream fragment were separately amplified by using the genomic DNA of *S. xylosus* C2a as template. The *ermB* gene was amplified from the pEC4 vector generating an amplicon of 1,366-bp. The three purified PCR products were annealed by overlapping PCR using the outside primers and inserted into pBT2 using appropriate restriction enzymes. The resulting plasmid was constructed in competent *E. coli* TOP10 and introduced into *S. xylosus* C2a by electroporation as described (Götz et al., 1983; Brückner, 1997). Gene replacement was allowed to take place by incubating the temperature-sensitive plasmid at 42°C as described (Brückner, 1997). Successful C2aΔ*nos* mutant was screened for resistance to erythromycin and sensitivity to chloramphenicol, indicating loss of the pBT2 backbone, and this was confirmed by PCR and sequencing.

**Table 2:** Oligonucleotides used in this study.

Name	Primer Sequence (5′ to 3′)^a,b^	Used for
Up923F1-EcoRI Up923R1-ErmF	CGGAATTCGACGCGTACCλTTCCATT **CACAATAGAGAGATGTCACC**TGTCGATλGGATTTCGCTTC	Amplification of sequence upstream *nos*
		
Dn923F2-ErmR Dn923R2-BamHI	**GGTATACTACTGACAGCTTCC**AACAGAAGCTACTGGTTGTCCA CGGGATCCGGTGTGTCTGCTTGGACλ	Amplification of sequence downstream *nos*
		
ErmF-Up923R1 ErmR-Dn923F2	**GAAGCGλTCCTTTATCGACA**GGTGACATCTCTCTATTGTG **TGGACAACCAGTAGCTTCTGTT**GGAAGCTGTCAGTAGTATACC	Amplification of *ermB*
		
Up923F3 Dn923R3	TCCTGCTCGCACATTACTTG CGTCAGGTATCTTGTTGCTCA	Control of *nos::erm* construction
		
Up923PF5-SalI Dn923R5-EcoRI	ACGCGTCGACGTGCTTGATAGCACATGλAAGGA CGGAATTCGGGTCGTTAATGλTGGACA	Complementation of *nos* in pRB473
		
Up923PF5-SalI Dn922-EcoRI	ACGCGTCGACGTGCTTGATAGCACATGλAAGGA CGGAATTCTTGCTCATTAGGTTGTGCTAATTC	Complementation of *nos-pdt* in pRB473
		
Up922-SalI Dn922-EcoRI	ACGCGTCGACGTAAGGGGTAACGTCλTGλ CGGAATTCTTGCTCATTAGGTTGTGCTAATTC	Complementation of *pdt* in pRB474
		
923F4 922R1	AAGCGλTCCTTTATCGACAC CTAATGGCAGGCCCAATAGA	Co-transcription *nos-pdt*
		
923F2 923R2	TGCAGAAGCGTTTGAATTTG GCTTCGATGCAGTGAGATGA	qRT-PCR of *nos* (SXYL_00923)
		
2505F 2505R	CGTCATCTTCACGλGTCATATTC CGCTAGTACACATTATTATCCAATAG	qRT-PCR of *katA* (SXYL_2505)
		
1551F 1551R	ATTCGTGGATTCGCATλAG AGCTTCTGGTAGTGACGT	qRT-PCR of *katB* (SXYL_1551)
		
2533F 2533R	TTCGATCATGAACGTATACCA GTGTCTGGTGAACCTTTAGAG	qRT-PCR of *katC* (SXYL_2533)
		
1303F 1303R	CGCAGCAGTAGAAGGAACTG ATGTCCACCGCCATTATTGC	qRT-PCR of the housekeeping gene *sod* (SXYL_1303) and control of DNA contamination in RNA extracts

*^a^Underlined regions represent restriction enzyme sequences.*

*^b^Primer containing overlapping end (in bold).*

### Creation of Complementation Plasmids

Complementation of the *nos* mutation was performed by amplifying the *nos* gene with its putative promoter. The corresponding amplicon was cloned into the shuttle vector pRB473 using appropriate enzymes, generating pRB*nos*. Similarly, an *nos-pdt* complementation vector (pRB*nospdt*) was constructed. A complementation vector pRB*pdt* was constructed, placing the expression of *pdt* under the constitutive promoter of pRB474. All PCR-amplified fragments were verified by sequencing. The resulting plasmids were constructed in competent *E. coli* TOP10 and introduced into *S. xylosus* C2aΔ*nos* by electroporation according to an established protocol (Brückner, 1997).

### Metmyoglobin Conversion and Assessment of Nitrosoheme Formation

A 20 mg/mL metmyoglobin solution was freshly prepared from equine heart myoglobin (Sigma-Aldrich) as described (Gündoğdu et al., 2006). Overnight *S. xylosus* cultures were inoculated in TSB supplemented with 2 mg/mL metmyoglobin to a final OD_600__nm_ of 0.5 (8 log CFU/mL). Cultures were incubated either under limited oxygenation (covered with mineral oil and without stirring) or under aerobic conditions as described above. After 24 h of incubation at 30°C, OD_600__nm_ and pH of the cultures were measured and cells were serial-diluted and plated on TSA for enumeration. Cultures were centrifuged and supernatants were used to measure the absorbance spectrum between 500 and 600 nm (BioMate 3, Thermo Fisher Scientific) using as control a solution of sterile TSB supplemented with metmyoglobin and incubated in the same conditions. Finally, supernatants were treated with acetone 1:4 (v/v) to extract the nitrosoheme from nitrosomyoglobin, and the absorbance spectrum was acquired between 450 and 640 nm (V-770 UV/visible, Jasco). Sterile TSB supplemented with metmyoglobin and extracted with acetone was used as control. Experiments were carried out in three independent biological replicates.

### Analysis of colony pigmentation

After overnight growth in TSB, *S. xylosus* strains were plated on TSA. After 2 days of growth (when pigmentation is strongest), 100 mg of each strain was collected from plates and pigments were extracted and quantified according the method of Morikawa et al. (2001). Briefly, cells were washed once in water, centrifuged for 5 min at 15,000 *g*, re-suspended in 1 mL of methanol and heated for 5 min at 55°C. Cells were centrifuged and the supernatant containing pigment was retrieved. The absorbance spectrum of methanol-extracted pigment was measured between 400 and 600 nm to evaluate carotenoid content. A peak at λ = 460 nm was detected and results are expressed as relative optical density at 460 nm with normalization to C2a. Experiments were performed with three independent biological replicates.

### Survival Experiments after Exposure to Different Stresses

The *S. xylosus* strains were grown in TSB under aerobic conditions. To assess sensitivity to oxidative stress, overnight cultures were diluted to an OD_600__nm_ of 0.05 with fresh medium and incubated until OD_600__nm_ was approximately 1. One milliliter of each culture was collected for serial dilutions and determination of colony-forming units (CFU)/mL on TSA plates. The remaining culture was treated with 150 mM hydrogen peroxide (Sigma-Aldrich) and incubated for 1 h at 30°C. 4,000 U/mL of catalase (Sigma–Aldrich) was added to quench residual H_2_O_2_ as described (Barrière et al., 2002). Cells were serial-diluted and plated in duplicate on TSA for enumeration of CFU from surviving cells.

To evaluate the impact of saline and acid stresses, overnight cultures of *S. xylosus* strains were freshly diluted (1/100) in TSB and grown under aerobic conditions at 30°C until OD_600__nm_ was approximately 0.3. Strains were diluted in NaCl-supplemented TSB (10 and 20% NaCl) or diluted in TSB with adjusted pH (5.0 and 6.0) to an OD_600__nm_ of 0.1 and incubated in 100-well microtiter plates with shaking at 30°C in a Bioscreen C plate reader, where turbidity was monitored every 30 min for 20 hours. Assays were performed with two independent cultures for each strain.

### Total RNA Extraction and Reverse Transcription

*Staphylococcus xylosus* strains were grown in TSB under aerobic conditions for 6 h and 24 h. Cell pellets were immediately frozen in liquid nitrogen to stabilize the bacterial RNA and stored at –80°C. For RNA extraction, cell pellets were thawed on ice and resuspended in 500 μL of cold Tris-EDTA buffer. Samples were transferred into tubes containing 600 mg zirconia-silica beads (0.1 mm diameter), 50 μL of sodium dodecyl sulfate (10%), 500 μL of acid phenol and 3.5 μL of β-mercaptoethanol. Cells were disrupted in a FastPrep^TM^ machine (MP Biomedicals). After addition of 200 μL of chloroform and centrifugation, the aqueous phase containing RNA was purified with the Nucleospin RNAII kit (Macherey Nagel) according to the manufacturer’s instructions. A supplementary treatment was performed with Turbo DNAse (Ambion) to remove residual DNA. Absence of contaminating DNA was checked by PCR targeting the *sod* gene (**Table 2**). Total RNA isolated was quantified using a Nanodrop 1000 (Thermo Fisher Scientific). RNA samples were stored at –80°C. RNA isolated from three independent biological replicates for each strain or condition (wild-type and its mutants at 6 and 24 h) was reverse-transcribed to cDNA with a SuperScript Reverse Transcriptase kit following the manufacturer’s instructions (Invitrogen).

### Evaluation of *nos-pdt* Co-transcription

To determine if the two adjacent genes, *nos* and *pdt*, were co-transcribed, cDNA from the mRNA species transcribed from C2a 24-h cultures was subject to PCR using a pair of primers targeting the *nos-pdt* junction region (**Table 2**). The conditions for the amplification were 5 min at 95°C, followed by 25 cycles of 30 s at 95°C, 30 s at 60°C and 120 s at 72°C and finally 5 min at 72°C. As controls, this amplification was also performed on C2a genomic template DNA and no template sample.

### Quantitative Real-time PCR

Expression of genes of interest was carried out by quantitative real-time PCR (qRT-PCR) using iQ^TM^ SYBR^®^ Green Supermix (Bio-Rad) and the MasterCycler RealPlex (Eppendorf). Thermal cycling consisted of 30 s at 95°C, followed by 40 cycles of 15 s at 95°C and 60 s at 60°C. The primers used are listed in **Table 2**. The relative fold change of gene expression, using measured *sod* housekeeping gene expression, was determined by the Livak (2^-ΔΔCt^) method (Livak and Schmittgen, 2001).

### Statistics

The data were analyzed by using GraphPad Prism software (version 5.01). The significance of experimental differences in bacterial growth, formation of myoglobin derivatives, and colony pigment production were analyzed by one-way analysis of variance (ANOVA) with Tukey’s multiple comparison.

## Results

### NOS Contributes to Growth under Aerobic Conditions

**FIGURE 1:**
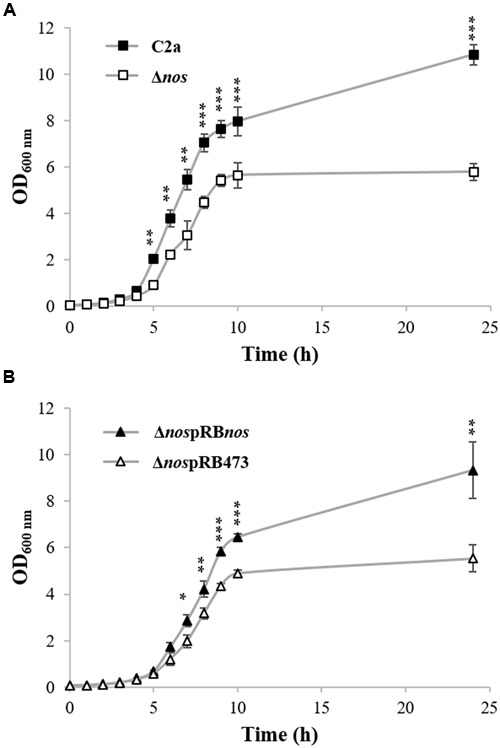
**Growth of *Staphylococcus xylosus* strains in TSB under aerobic conditions. (A)** *S. xylosus* C2a and Δ*nos*. **(B)** Δ*nos*pRB*nos* and Δ*nos*pRB473 with chloramphenicol. Data represent means ± SD from *n* = 3 independent biological replicates. *p < 0.05; **p < 0.01; ***p < 0.001.

To define the role of NOS activity in *S. xylosus* C2a, a mutant strain (Δ*nos*) was created by insertion of an erythromycin resistance gene cassette into the *nos* coding sequence. Deletion of the *nos* gene was confirmed by PCR analysis and sequencing (data not shown). When grown in TSB under aerobic conditions, the Δ*nos* mutant displayed a slight growth defect compared with the wild type (**Figure 1A**). Complementation of the Δ*nos* mutant with a plasmid expressing the *nos* gene (Δ*nos*pRB*nos*) showed restored growth (**Figure 1B**). The wild type and the Δ*nos* and *nos* complemented mutants did not differ in growth in microtiter plates under microaerobic conditions (**Supplementary Figure S1**).

### PDT But Not NOS Is Required for Growth in Minimal Medium

**FIGURE 2:**
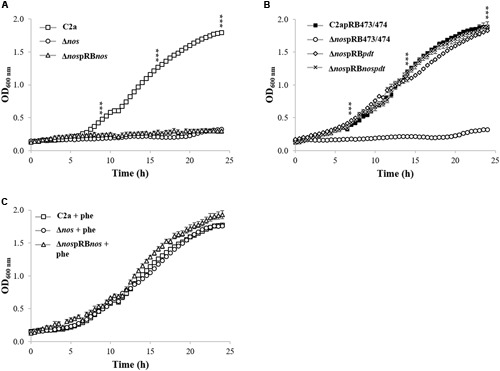
**Growth of *S. xylosus* strains in chemically defined minimal medium in the Bioscreen Assay. (A)** Growth of *S. xylosus* C2a, Δ*nos*, and Δ*nos*pRB*nos*, **(B)** Growth of C2a, Δ*nos* and Δ*nos*pRB*nos* with phenylalanine (phe), **(C)** Growth of C2apRB473 (identical growth as C2apRB474), Δ*nos*pRB473 (identical growth as Δ*nos*pRB474), Δ*nos*pRB*pdt* and Δ*no*spRB*nospdt*. Data represent means ± SD from *n* = 3 independent biological replicates. ***p < 0.001.

Growth was measured in minimal medium under microaerobic conditions for the wild-type C2a, the Δ*nos* mutant and the *nos* complemented Δ*nos* mutant (**Figure 2A**). The Δ*nos* mutant was not able to grow in this minimal medium and growth was not restored by plasmid complementation with the *nos* gene (**Figure 2A**). Since the *nos* gene is in a cluster with the *pdt* gene, we tested the hypothesis that both genes were co-transcribed (**Supplementary Figure S2**). RT-PCR experiments were carried out using a primer annealing specifically with *nos* and a primer annealing specifically with *pdt* (**Table 2**). Amplification of a 1,421-bp fragment indicated a co-transcription of both *nos* and *pdt* (data not shown). We therefore speculated that the deletion of *nos* and the insertion of *ermB* had a polar effect on the downstream gene *pdt*, leading to a frameshift in the *pdt* open reading frame (ORF). The *pdt* gene encodes a prephenate dehydratase (EC4.2.1.51), which catalyzes the penultimate reaction in the phenylalanine biosynthesis. Supplementation with phenylalanine restored the growth of the Δ*nos* and the *nos* complemented (Δ*nos*pRB*nos*) mutants (**Figure 2B**), while no restoration of growth was observed with other amino acids such as tryptophan or tyrosine, which, like phenylalanine, are derived from chorismate (data not shown). Moreover, complementation of the Δ*nos* mutant with *pdt* (Δ*nos*pRB*pdt*) or with the *nos*-*pdt* operon (Δ*nos*pRB*nospdt*) restored growth to the wild-type C2a level (**Figure 2C**).

### NOS Contributes to NO Production

**FIGURE 3:**
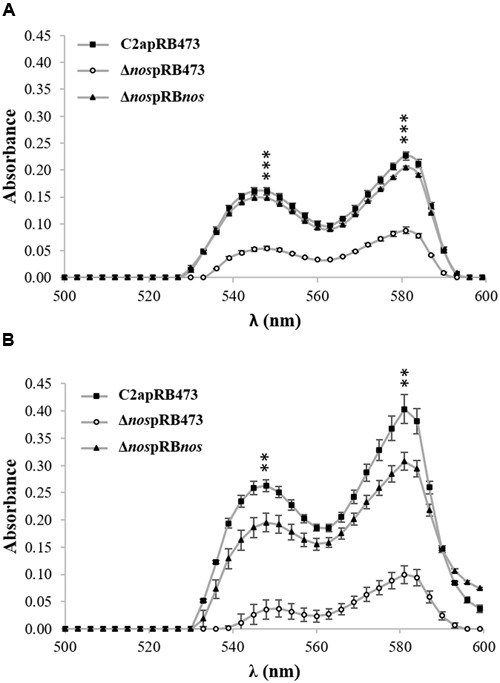
**Formation of red myoglobin derivatives.** *S. xylosus* C2apRB473, Δ*nos*pRB473 and Δ*nos*pRB*nos* were incubated in TSB supplemented with metmyoglobin (2 mg/mL) under **(A)** limited oxygenation (no stirring and reaction mixture covered with mineral oil) and **(B)** aerobic conditions (150 rpm). Formation of red pigment was evaluated by measuring absorbance. Data represent means ± SD from *n* = 3 independent biological replicates. **p < 0.01; ***p < 0.001 at λ_548__nm_ and λ_581__nm._

The formation of the red myoglobin derivatives occurred for *S. xylosus* C2a under limited oxygenation (**Figure 3A**) and aerobic conditions (**Figure 3B**), where different forms of myoglobin came from the conversion of metmyoglobin (brown) to some red derivatives, such as oxymyoglobin and nitrosomyoglobin. The red pigment content was sharply decreased, but not completely abolished, in the Δ*nos* mutant compared with the wild type, and was restored to the wild-type level in the *nos* complemented Δ*nos* mutant under both conditions (**Figure 3**). Under limited oxygenation after 24 h of incubation, the cellular population was close to initial level, 8 log CFU/mL, for C2a (7.8 ± 0.04), Δ*nos* mutant (7.6 ± 0.40) and *nos* complemented Δ*nos* mutant (7.2 ± 0.01). Under aerobic conditions, the red pigment level was higher (**Figure 3B**) and the cellular population reached 9 log CFU/mL for C2a (9.1 ± 0.04), Δ*nos* mutant (9.2 ± 0.20) and Δ*nos* complemented mutant (9.2 ± 0.10).

**FIGURE 4:**
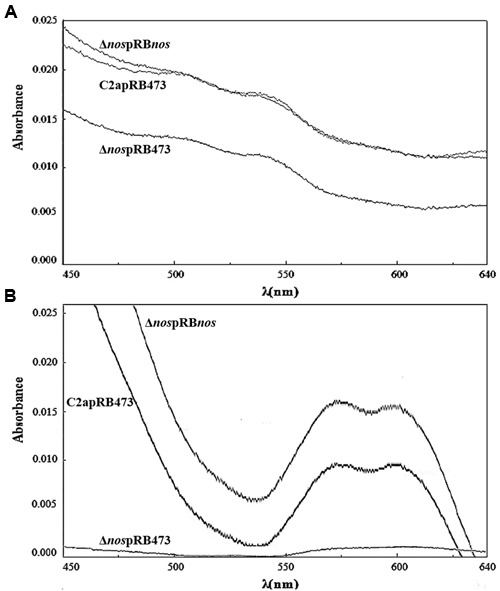
**Evidence of nitrosoheme.** Nitrosoheme was extracted from nitrosomyoglobin with 80% acetone from supernatants of strains grown in TSB supplemented with metmyoglobin and inoculated under **(A)** limited oxygenation (no stirring and reaction mixture covered with mineral oil) and **(B)** aerobic conditions (150 rpm). Curves show spectra representative of three biological replicates.

To estimate the production of nitrosomyoglobin, the nitrosoheme of culture supernatants from *S. xylosus* C2a and its mutants was extracted and measured (**Figure 4**). Under limited oxygenation (**Figure 4A**), where the pH of cultures was about 6.0, the nitrosoheme formation of the Δ*nos* mutant was reduced (A_540nm_ = 0.060 ± 1.1E-03) compared with the wild type (A_540__nm_ = 0.090 ± 7.1E-04) and the *nos* complemented Δ*nos* mutant (A_540__nm_ = 0.090 ± 1.1E-03). Under aerobic conditions (**Figure 4B**), where the pH of the cultures was about 8.0, maximum absorption shifted, as described (Yu et al., 2016), to λ_580__nm_ instead of λ_540__nm_. In these conditions, no nitrosoheme was formed for the Δ*nos* mutant (A_580__nm_ < 0.005) compared with the wild type (A_580__nm_ = 0.060 ± 2.8E-03) and the *nos* complemented Δ*nos* mutant (A_580nm_ = 0.080 ± 1.4E-03).

The Δ*nos* mutant and the *pdt* complemented Δ*nos* mutant had the same activity, while for the *nospdt* complemented Δ*nos* mutant the formation of red myoglobin derivatives was restored to the wild-type level (data not shown). These results clearly demonstrated the contribution of NOS and not PDT to the production of NO in *S. xylosus* C2a.

### NOS Affects Colony Pigmentation

**FIGURE 5:**
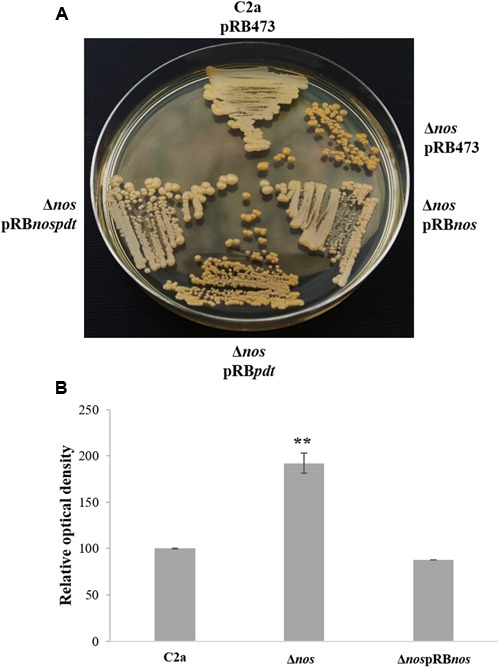
**Colony pigment production by *S. xylosus* strains. (A)** *S. xylosus* C2apRB473, Δ*nos*pRB473, Δ*nos*pRB*nos*, Δ*nos*pRB*pdt* and Δ*nos*pRB*nospdt* streaked on Tryptic Soy Agar (TSA) plates containing chloramphenicol for three days. **(B)** Measurement of pigmentation after methanol extraction at λ_460__nm_. The relative optical density is normalized to C2a, which was set at 100. Data represent means ± SD from *n* = 3 independent biological replicates. **p < 0.01.

When grown on TSA plates, the Δ*nos* mutant displayed enhanced pigmentation relative to the wild type. This pigmentation returned to wild-type level upon complementation with the *nos* and *nos-pdt* genes, but not with the *pdt* gene alone (**Figure 5A**). Pigments of the wild type and the Δ*nos* and Δ*nos*pRB*nos* mutants were extracted and absorbance at 460 nm was measured. Production of carotenoid pigment in the Δ*nos* mutant was significantly higher than in the wild type, and returned to the wild-type level in the *nos* complemented mutant (**Figure 5B**). These results clearly demonstrated that solely NOS was involved in the pigmentation phenotype. This difference in carotenoid pigment production between the wild type and the Δ*nos* mutant was, however, not observed with the bacterial pellets after liquid cultures in TSB, even after prolonged growth for several days (data not shown).

### NOS Protects Specifically against Peroxide Stress

**FIGURE 6:**
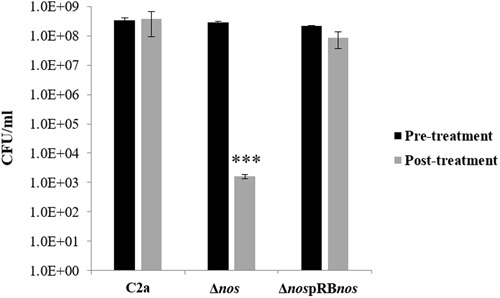
**Effect of *nos* deletion on sensitivity to oxidative stress.** *S. xylosus* C2a, Δ*nos*, and Δ*nos*pRB*nos* strains were grown under aerobic conditions up to OD = 1 and treated for 1 h with 150 mM hydrogen peroxide (H_2_O_2_). Cells, before and after treatment, were enumerated. Data represent means ± SD from *n* = 2 independent biological replicates. *p < 0.05.

Stress challenge assays with 150 mM H_2_O_2_ were performed on the wild-type C2a and the Δ*nos* and *nos* complemented Δ*nos* mutants to investigate a possible oxidative stress-sensitive phenotype in the Δ*nos* mutant. After 1-h treatment, the Δ*nos* mutant presented a significant reduction in cell viability (5 log) compared with the wild type (**Figure 6**). The phenotype was restored to the wild-type level in the *nos* complemented mutant (**Figure 6**). To evaluate the specificity of NOS in protecting against oxidative stress, stress challenge assays in saline and acid conditions were performed on the wild type and the Δ*nos* and *nos* complemented Δ*nos* mutants (**Supplementary Figure S3**). As the salt concentration increased (**Supplementary Figure S3A**) or the pH decreased (**Supplementary Figure S3B**), bacterial growth was affected and decreased for all strains with no significant differences between strains. These results demonstrated that NOS was not required to overcome those stresses.

### NOS Modulates Catalase Gene Expression

**FIGURE 7:**
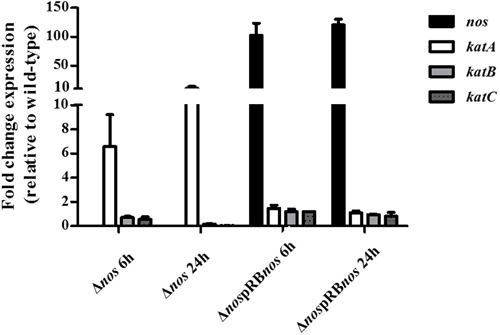
**Expression of *nos* and *kat* genes.** *S. xylosus* C2a, Δ*nos*, and Δ*nos*pRB*nos* strains were grown in TSB under aerobic conditions for 6 and 24 h. Expression of *nos* and *kat* genes was evaluated by qRT-PCR. The Livak method (2^-ΔΔCt^) was applied to analyze expression, using *sod* as reference gene. Results are expressed as means ± SD from *n* = 3 independent biological replicates.

To determine whether increased H_2_O_2_-sensitive phenotype and defects in growth under aerobic conditions of the Δ*nos* mutant were due to differential expression of catalase genes, we evaluated expression of the *katA*, *katB*, and *katC* genes by qRT-PCR. The *katA* gene was overexpressed 6- and 10-fold at 6 h and 24 h, respectively in the Δ*nos* mutant related to the wild type (**Figure 7**). Expression of the two genes *katB* and *katC* was not modified in the Δ*nos* mutant at 6 h and was sharply downregulated at 24 h by comparison with the wild type (**Figure 7**). In the *nos* complemented mutant, only the *nos* gene was highly overexpressed at the two times of incubation, while the expression of the three catalase genes was not modified by comparison with the wild type. However, there was no difference in survival between the wild type and the *nos* complemented Δ*nos* mutant when treated with 200 mM H_2_O_2_ (4 log reduction) and 250 mM (6 log reduction).

## Discussion

*Staphylococcus xylosus* is commonly used as a starter culture for fermented meat products, mainly because it reduces nitrate to nitrite, which undergoes chemical reactions leading to NO, which is responsible for color development (Talon and Leroy, 2006). As the safety of nitrite as a food additive is questioned, we studied the ability of *S. xylosus* to produce NO *via* a pathway catalyzed by NOS. To demonstrate the production of NO by *S. xylosus*, we used the property of NO to form a NO complex of Fe(II) myoglobin, called nitrosomyoglobin. In previous studies *S. xylosus* FAX-1 and *S. xylosus* A1 were able to convert metmyoglobin to nitrosomyoglobin *in vitro* and in raw meat batter (Morita et al., 1998; Li et al., 2013, 2016). However, the mechanism of such NO production was not established. Here we demonstrate that NOS activity of *S. xylosus* led to production of NO. Under limited oxygenation, the Δ*nos* mutant remained able to form nitrosoheme (about 35-40% related to the wild-type level). The Δ*nos* mutant was unable to produce NO under aerobic conditions. The loss of NOS activity in *S. aureus* UAMS-1 mutant led to NO production of 65% as evaluated using fluorescent stain DAF-FM diacetate after growth on agar plates (Sapp et al., 2014), while the deletion of *nos* in *S. aureus* UAMS1182 completely abolished NO production after growth under aerobic conditions (van Sorge et al., 2013). These results revealed that some *Staphylococcus* strains are able to produce NO by other pathways under limited oxygenation. NO production can be achieved *via* molybdoenzymes, which are recognized for their ability to catalyze reduction of nitrite to NO radical (Maia and Moura, 2015). The molybdenum-containing respiratory nitrate reductase is believed to be an important source of NO (Maia and Moura, 2015). *S. xylosus* C2a has a nitrate reductase that was previously shown to be expressed and active under static conditions (Talon et al., 1999). The involvement of NOS under aerobic conditions seems relevant to the oxygen requirement of NOS activity (Crane et al., 2010). The potential for NOS activity has been investigated in 86 strains belonging to 17 CNS species (Sánchez-Mainar et al., 2014). Only one strain of *S. haemolyticus* showed NOS activity, based on its ability to produce L-citrulline in a meat-simulating medium supplemented with arginine under aerobic conditions (Sánchez-Mainar et al., 2014).

The deletion of *nos* in *S. xylosus* C2a slightly affected growth in complex medium under aerobic but not under microaerobic conditions. This *nos* deletion also increased H_2_O_2_ susceptibility. Such results are in accordance with previous studies showing that NO production through NOS activity protects *Bacillus* and *S. aureus* against oxidative stress generated by H_2_O_2_ exposure (Gusarov and Nudler, 2005; Shatalin et al., 2008; van Sorge et al., 2013). In *B. subtilis*, exogenous or endogenous NO protected cells from oxidative stress by boosting the activity of KatA, the major catalase (Gusarov and Nudler, 2005). Also, while *sodA* expression increased sharply in wild-type cells of *B. subtilis* during exponential growth, it was abolished in the Δ*nos* mutant (Gusarov et al., 2009). In *S. xylosus* C2a Δ*nos* mutant, the loss of NOS activity resulted in modulation of the expression of genes encoding catalases with upregulation of *katA* and downregulation of *katB* and *katC*. We showed in a previous study that *S. xylosus* responded to nitrosative stress generated by nitrite in a meat model, notably by the upregulation of *katB* and *katC* under the probable control of the repressor PerR and the downregulation of *katA*, which was not under PerR control (Vermassen et al., 2014). In this study, the limited amount of endogenous NO produced by the Δ*nos* mutant may not derepress PerR, contributing to the downregulation of *katB* and *katC*. In contrast to *B. subtilis*, expression of the gene *sodA* was not modified in the *S. xylosus*Δ*nos* mutant (*sod* was used as housekeeping gene in our study).

The *S. xylosus* C2a Δ*nos* mutant displayed higher colony pigmentation than the wild-type strain after prolonged cultivation on agar medium, as observed for the *S. aureus*Δ*nos* strain UAMS-1 (Sapp et al., 2014). *S. aureus* produced the intermediary yellow carotenoid 4,4′-diaponeurosporene, which, after prolonged cultivation, is converted to the yellow-orange end-product staphyloxanthin (Wieland et al., 1994). The yellow pigment in *S. xylosus* is likely to be a carotenoid pigment with peak absorbance at 460 nm. The carotenoid pigment of *S. aureus* protects against reactive oxygen species, so a non-pigmented mutant is more susceptible to oxidative killing (Clauditz et al., 2006). The increased colony pigmentation of the *S. xylosus* C2a Δ*nos* mutant could be a mechanism to cope with oxidative stress in conditions of extended incubation only on agar medium, as no pigmentation was observed in liquid medium.

The *S. aureus nos* gene is co-transcribed with the *pdt* gene encoding a prephenate dehydratase, which catalyzes the penultimate reaction in phenylalanine biosynthesis (Sapp et al., 2014). This genetic arrangement is present in all publically available staphylococcal genome sequences and seems to be unique to the *Staphylococcus* genus (Sapp et al., 2014). As anticipated, co-transcription of the *nos*-*pdt* cluster was also demonstrated in *S. xylosus*. However, the role of the NOS and PDT enzymes of *S. xylosus* C2a did not appear to be interlinked. PDT was essential for *S. xylosus* growth only in the absence of phenylalanine, while NOS was not required to sustain phenylalanine biosynthesis.

## Conclusion

Our results demonstrate NOS-dependent NO production in a coagulase-negative *Staphylococcus*. This endogenous NO production contributed to growth under aerobic conditions and to the cytoprotective effect against oxidative stress. *S. xylosus* is a species usually used as a starter culture in meat products. Therefore, NOS-dependent NO production in *S. xylosus* needs to be further characterized in meat products and optimized as a potential alternative to nitrate and nitrite in these products.

## Author Contributions

SL and RT conceived the study. GR, SL and RT designed the experiments and wrote the manuscript. GR performed the laboratory experiments. GR, VZ, PD, TS, SL and RT analyzed data. All the authors contributed to preparing the final version of the manuscript, read and approved the final manuscript.

## Conflict of Interest Statement

The authors declare that the research was conducted in the absence of any commercial or financial relationships that could be construed as a potential conflict of interest.

## Supplementary Material

FIGURE S1 ***Staphylococcus xylosus* strains in the Bioscreen Assay**. *S. xylosus* C2a, Δ*nos*, and Δ*nos*pRB*nos* in TSB. Data represent means ± SD from *n* = 3 independent biological replicates.

FIGURE S2 **Genetic cluster *nos-pdt* of *S. xylosus* C2a**. The cluster of the two genes *nos-pdt* is depicted with primers used for PCR co-transcription.

FIGURE S3 **Impact of stresses on growth under microaerobic conditions of *S. xylosus* strains. (A)** Impact of salt and **(B)** impact of acid stresses. Data represent means ± SD from *n* = 3 independent biological replicates.
