# Supplementary figures and images for: Evidence for Nitric Oxide Synthase Activity in Staphylococcus xylosus Mediating Nitrosoheme Formation
Source: Front Microbiol. 2017 Apr 6;8:598. doi: 10.3389/fmicb.2017.00598 (PMC5382197; doi:10.3389/fmicb.2017.00598)

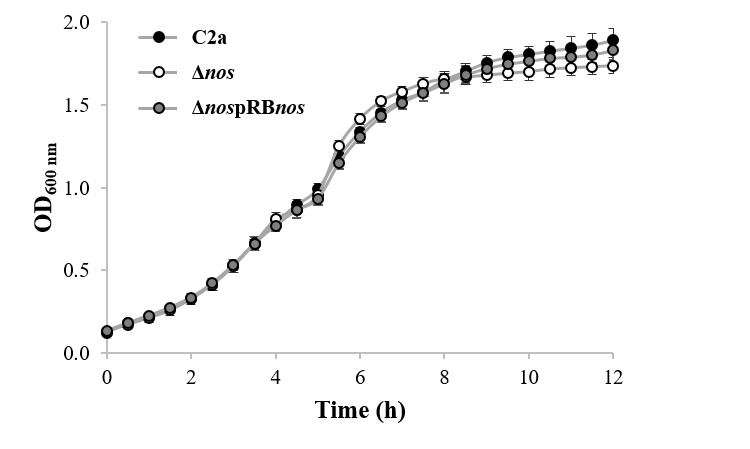

Supplement: FIGURE S1 — Staphylococcus xylosus strains in the Bioscreen Assay. S. xylosus C2a, Δnos, and ΔnospRBnos in TSB. Data represent means ± SD from n = 3 independent biological replicates. [file Image_1.TIF]

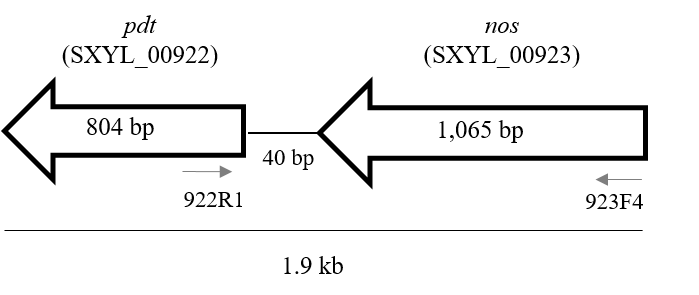

Supplement: FIGURE S2 — Genetic cluster nos-pdt of S. xylosus C2a. The cluster of the two genes nos-pdt is depicted with primers used for PCR co-transcription. [file Image_2.TIF]

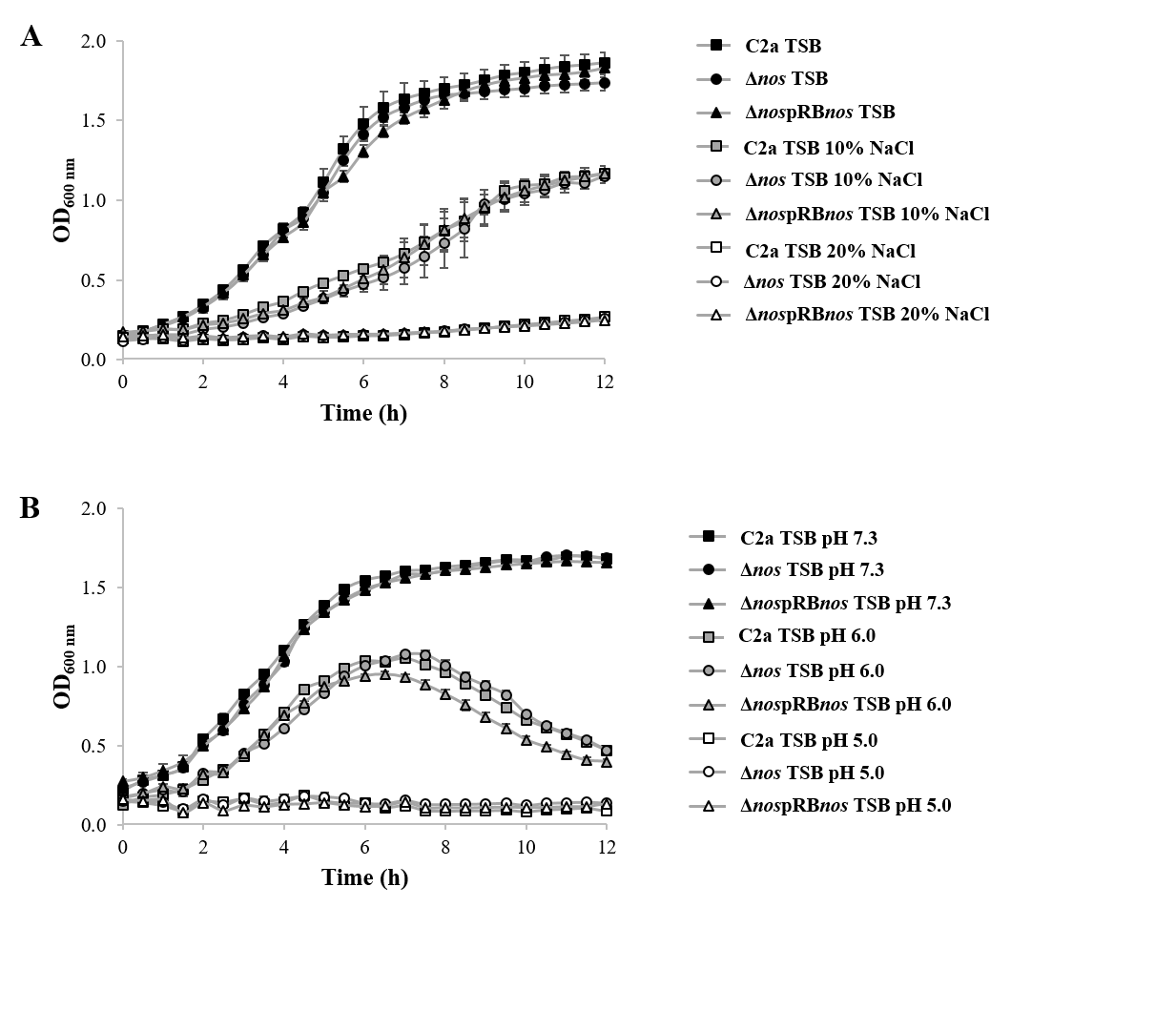

Supplement: FIGURE S3 — Impact of stresses on growth under microaerobic conditions of S. xylosus strains. (A) Impact of salt and (B) impact of acid stresses. Data represent means ± SD from n = 3 independent biological replicates. [file Image_3.TIF]
